# MicroRNA-18a-5p Administration Suppresses Retinal Neovascularization by Targeting FGF1 and HIF1A

**DOI:** 10.3389/fphar.2020.00276

**Published:** 2020-03-10

**Authors:** Ji-Tian Guan, Xin-Xin Li, De-Wei Peng, Wen-Meng Zhang, Jia Qu, Fan Lu, Robert J. D’Amato, Zai-Long Chi

**Affiliations:** ^1^State Key Laboratory of Ophthalmology, Optometry and Visual Science, Eye Hospital of Wenzhou Medical University, Wenzhou, China; ^2^International Joint Research Center for Regenerative Medicine and Neurogenetics, Wenzhou Medical University, Wenzhou, China; ^3^Vascular Biology Program, Department of Surgery, Boston Children’s Hospital, Boston, MA, United States; ^4^Department of Ophthalmology, Harvard Medical School, Boston, MA, United States

**Keywords:** miR-18a-5p, neovascularization, proliferative retinopathy, FGF1, HIF1A

## Abstract

Pathologic ocular neovascularization commonly results in visual impairment or even blindness in numerous fundus diseases, including proliferative diabetic retinopathy (PDR), retinopathy of prematurity (ROP), and age-related macular degeneration (AMD). MicroRNAs regulate angiogenesis through modulating target genes and disease progression, making them a new class of targets for drug discovery. In this study, we investigated the potential role of miR-18a-5p in retinal neovascularization using a mouse model of oxygen-induced proliferative retinopathy (OIR). We found that miR-18a-5p was highly expressed in the retina of pups as well as retinal endothelial cells, and was consistently down-regulated during retinal development. On the other hand, miR-18a-5p was increased significantly during pathologic neovascularization in the retinas of OIR mice. Moreover, intravitreal administration of miRNA mimic, agomiR-18a-5p, significantly suppressed retinal neovascularization in OIR models. Accordingly, agomir-18a-5p markedly suppressed human retinal microvascular endothelial cell (HRMEC) function including proliferation, migration, and tube formation ability. Additionally, we demonstrated that miR-18a-5p directly down-regulated known vascular growth factors, fibroblast growth factor 1 (FGF1) and hypoxia-inducible factor 1-alpha (HIF1A), as the target genes. In conclusion, miR-18a-5p may be a useful drug target for pathologic ocular neovascularization.

## Introduction

The intraretinal vasculature supplies the inner part of the retina with oxygen and nutrients (Fruttiger, 2007). In mice, retinal vasculature development begins around birth and is completed in the third postnatal week (Stahl et al., 2010; Selvam et al., 2018). Dysfunctional angiogenesis is involved in many diseases, including cardiovascular diseases, tumorigenesis, and proliferative retinopathies (Folkman, 1995). Proliferative retinopathies such as proliferative diabetic retinopathy (PDR), retinopathy of prematurity (ROP), and retina vein occlusion (RVO) are characterized by pathological retinal neovascularization, which is the leading cause of blindness (Augustin and Koh, 2017). Retinal neovascularization is a very complicated pathophysiologic process, which is driven by the proangiogenic factors, such as vascular endothelial growth factor (VEGF), fibroblast growth factor (FGF), and erythropoietin (Liu et al., 2016). Currently, the clinical treatments for proliferative retinopathy include laser photocoagulation, vitrectomy surgery, and anti-VEGF drugs. Laser photocoagulation, the standard treatment for proliferative retinopathy, has a high rate of visual and anatomic success (Wallace and Wu, 2013). Anti-VEGF drugs are widely used as the major treatment in both ROP and DR to achieve certain efficacy (Xu et al., 2018). However, despite these considerable advances, an increasing number of clinical studies have reported that laser treatment and anti-VEGF therapy does have some limitations and causes undesirable side effects (Potente et al., 2011; Xu et al., 2018). Therefore, it is critical to explore and identify additional factors that regulate pathological neovascularization in order to exploit new therapeutic drugs.

MicroRNAs (miRNAs) are small endogenous non-coding RNAs composed of 21–25 nucleotides that negatively regulate gene expression at the posttranscriptional level by mediating mRNA degradation and/or translational repression (He and Hannon, 2004; Bartel, 2009). MiRNAs have been reported to play a key role in retinal vascular development and disease progression (Shen et al., 2008; Sundermeier and Palczewski, 2012). Several miRNAs were identified to inhibit retinal neovascularization, such as miR-126, miR-150, miR-223, and miR-384-3p (Shi et al., 2013; Liu et al., 2015; Zhou et al., 2016; Xia et al., 2018). Until now, several miRNA mimics or inhibitors have been tested in clinical trials for treatment of various diseases (Bader, 2012; Janssen et al., 2013; van Zandwijk et al., 2017). There will undoubtedly be many more breakthroughs in the development of novel miRNA-based therapeutics for the treatment of neovascular eye diseases.

The miR-17-92 cluster is one of the first miRNAs found to be associated with tumor angiogenesis. This conserved miRNA cluster encodes miR-17, miR-18a, miR-19a/b, miR-20a, and miR-92a and is highly expressed in tumors (Kaluza et al., 2013). Several studies have proven that the miR-17-92 cluster serves an antiangiogenic role by targeting the proangiogenic factors (Bonauer et al., 2009; Nunes et al., 2015). MiR-18a and miR-19a have been shown to repress thrombospondin 1 (TSP1) and connective tissue growth factor (CTGF) (Dews et al., 2006; Chamorro-Jorganes et al., 2016). Ferreira et al. found that argonaute-2 can promote miR-18a entry in human brain endothelial cells (ECs), and miR-18a can modulate the EC proliferation and migration derived from human cerebral arteriovenous malformation (AVM), and produce aberrant tubule structure compared with normal brain ECs (Ferreira et al., 2014a, b). However, it is not completely understood whether miR-18a is altered in pathologic ocular neovascularization or whether it is useful as a new molecular target for the treatment of neovascular eye diseases.

Currently, the oxygen-induced retinopathy (OIR) mouse model has been widely used in studies ROP and PDR (Smith et al., 1994; Connor et al., 2009) to evaluating the efficacy of antiangiogenic agents. Neonatal mice are exposed to high oxygen levels (75% oxygen) from postnatal day 7 (P7) for 5 days. Hyperoxia inhibits retinal vessel growth, and relatively hypoxia (removed to room air) triggers both vascular regrowth and neovascularization. In this study, we investigated the effects of miR-18a-5p on the human retinal microvascular endothelial cell (HRMEC) function and OIR models to elucidate the participation of miR-18a-5p in the initiation and progression of ocular neovascularization. Moreover, we explored the mechanism underlying miR-18a-5p activity in pathological angiogenesis through screening and validating the specific miR-18a-5p target genes. Taken together, these results might provide a reliable theoretical foundation to improve the treatment of neovascular ocular diseases.

## Materials and Methods

### High-Throughput Sequencing

C57BL/6J mice were obtained from Beijing Vital River Laboratory Animal Technology Co., Ltd., Total RNA was extracted from fresh retinas isolated from mice in different developmental periods: postnatal day 1 (P1), P7, and P17. Briefly, the mice were euthanized, and retinas were isolated from the dissected eye and moved into the RNA-Solv Reagent (Omega Bio-tek, Norcross, GA, United States). T10 basic S25 ULTRA-TURRAX Disperser (IKA, Guangzhou, China) was used to homogenize retinas. Total RNA was extracted by miRNA Kit (Omega Bio-tek) according to the manufacturer’s instructions. RNA quantity and quality were assessed by spectrophotometer 1510 (Thermo Fisher Scientific, Waltham, MA, United States). MiRNA libraries construction and sequencing on Illumina HiSeq 2500 (San Diego, CA, United States) were performed by RiboBio Co., Ltd. (Guangzhou, China). All animal experiments were performed strictly according to the ARVO Statement for the Use of Animals in Ophthalmic and Vision Research and approved by the Animal Care and Use Committee of Wenzhou Medical University.

### Sequencing Data Analysis

For miRNA data analysis, clean reads were obtained from the 50-nt raw reads by removing the adaptor sequences and discarding low-quality reads. HISAT2 (Kim et al., 2015) was used to align the clean reads to the mouse reference genome mm10 with default parameters. Differential expression was assessed by DEseq using RPM (the number of reads per million) value as input. Differentially expressed miRNAs were chosen according to the criteria of fold change >2 and adjusted *P* < 0.05. All the differentially expressed miRNAs were used for temporal expression pattern analysis by STEM (Ernst and Bar-Joseph, 2006). Typical downregulated and upregulated miRNAs were clustered using the Amazing Heat Map function of TB tools (Chen et al., 2018). The differentially expressed miRNAs were clustered based on Euclidean distance using average linkage clustering with Cluster 3.0 software (Eisen Lab, University of California at Berkeley, United States). TreeView (Eisen Lab, University of California at Berkeley, United States) was used to visualize the clustered heat map.

### OIR Mouse Model and Retinal Neovascularization Quantification

C57BL/6J mice were used to generate the oxygen-induced proliferative retinopathy (OIR) model as previously described (Smith et al., 1994; Connor et al., 2009). Newborn mice and their nursing mother mice were exposed to 75% oxygen at P7 and returned to room air at P12. Mice were sacrificed at P17, followed by retina dissection and staining with Isolectin GS-IB_4_ (Life Technologies, Eugene, OR, United States). The pups kept in room air throughout the experiments were used as the control. Intravitreal injection was conducted following previous protocols (Bai et al., 2011; Fu et al., 2017). Agomir-18a-5p at a dose of 1.5 μg (RiboBio, Guangzhou, China) diluted in two microliter of phosphate-buffered saline (PBS, Biological Industries, Beit Haemek, Israel) was intravitreously injected into the eye of OIR mice at P12 using a 33-gauge needle (Hamilton, Reno, NV, United States). The contralateral eye injected with scrambled agomir diluted in PBS was used as the negative control (NC). Neovascularization (neovascular tuft) and vaso-obliteration in OIR were quantified by using Adobe Photoshop and ImageJ software. Quantification was performed with the identity of the samples masked, with n being the number of mice quantified.

### Retinal Endothelial Cells (RECs) Isolation

The retinal cell suspension of mice was prepared using a modified method according to Su et al. (2003). Briefly, the retinas were dissected out from the mice eyes (6 to 7 pups from one litter), and minced into small pieces in Hanks’ Balanced Salt Solution (HBSS; Thermo Fisher Scientific). Following digestion with collagenase type I (1 mg/ml) in Dulbecco’s modified Eagle medium (DMEM, Thermo Fisher Scientific) for 30–45 min at 37°C, the cellular digests were filtered through the 40 μm nylon mesh. DMEM with 10% fetal bovine serum (FBS; Thermo Fisher Scientific) was then added and centrifuged at 400 × *g* for 10 min to pellet cells. The cells were resuspended and incubated with anti-CD31 MicroBeads (MicroBeads conjugated to monoclonal anti-mouse CD31 antibodies; Miltenyi Biotec GmbH, Bergisch Gladbach, Germany). Then, the cell suspension is loaded onto a MACS Column, which is placed in the magnetic field of a MACS Separator (Miltenyi Biotec GmbH). The magnetically labeled cells were washed and flushed out with the appropriate amount of PBS containing 0.5% bovine serum albumin (BSA) and 2 mM EDTA according to the manufacturer’s protocol. The collected cells were plated in a 8.5 μg/ml of Bovine Plasma Fibronectin (BPF; EMD Millipore) pre-coated 24-well plate and incubated in Endothelial Cell Medium (ECM; ScienCell, Carlsbad, CA, United States) containing 5% FBS, 20 μg/ml of Endothelial Cell Growth Supplement (ECGS; EMD Millipore, Temecula, CA, United States), 100 μg/ml streptomycin, and 100 U/ml penicillin at 37°C with 5% CO_2_. Immunofluorescence (IF) staining was conducted to identify the RECs with anti-CD31 (BD Bioscience) and anti-VE-cadherin (CST, Beverly, MA, United States) antibodies.

### Real-Time PCR

TRIzol Reagent (Thermo Fisher Scientific) was used to extract total RNA of retinas isolated from mice. Meanwhile, total RNA was extracted from the isolated RECs. To measurement of miR-18a-5p levels, real-time polymerase chain reaction (PCR) was performed using a miDETECT A Track miRNA qRT-PCR Starter Kit (RiboBio) according to the manufacturer’s protocol. Total RNA was added multiple Poly (A) using Poly (A) Polymerase, and reverse transcribed to cDNA using RTase mix and miDETECT A Track Uni-RT Primer. Real-time PCR was carried out on QuantStudio 5 Real-Time PCR Systems (Applied Biosystems, Foster City, CA, United States) using 2 × SYBR Green Mix with miDETECT A Track miRNA-18a-5p Forward Primer. U6 was used as the control.

To detect target genes mRNA levels, total RNA was reversely transcribed to cDNA using the Reverse Transcription System (Promega, Madison, WI, United States). Real-time PCR was carried out using iTaq Universal SYBR Green Supermix (Bio-Rad, Hercules, CA, United States). GAPDH served as the internal control. All primers were synthesized by Invitrogen (Shanghai, China). The primer sequences used in this study were as follows: FGF1: 5′-ACACCGACGGGCTTTTATACG-3′ (forward), 5′-CCCATTCTTCTTGAGGCCAAC-3′ (reverse); HI F1A: 5′-TGTAATGCTCCCCTCACCCA-3′ (forward), 5′-TGCA GGGTCAGCACTACTTC-3′ (reverse); GAPDH: 5′-ATCGTGG AAGGACTCATGACCACA-3′ (forward), 5′-AGAGGCAGGGA TGATGTTCTGGA-3′ (reverse). Each sample was detected in triplicate, and the specificity of PCR reaction was estimated by melt curve. Fold-change of miRNA level was calculated using the ΔΔCT method.

### Cell Culture

HRMECs were obtained from Angio-Proteomie (Peabody, MA, United States). HRMECs were cultured in endothelial cell medium (ECM) supplemented with 5% Newborn Calf Serum (NCS; Life Technologies), 20 μg/ml of ECGS, 100 μg/ml streptomycin, and 100 U/ml penicillin in a T-25 flask coated with 8.5 μg/ml of BPF at 37°C with 5% CO_2_. When the cells became 90% confluent, they were subcultured at a 1:3 ratio. HEK293 cells were purchased from the American Type Culture Collection (ATCC, Manassas, VA, United States) and cultured in DMEM supplemented with 10% NCS and incubated at 37°C with 5% CO2.

### Proliferation Assay

Proliferation of HRMECs was detected by Cell Counting Kit-8 (CCK-8, Dojindo Molecular Technologies, Shanghai, China) assay. Cells were seeded in 96-well plates (3 × 10^3^/well). Agomir-18a-5p (50 nM) or a scrambled negative control (NC) was transfected into HRMECs using Lipofectamine RNAiMAX Reagent (Invitrogen, Carlsbad, CA) following the manufacturer’s instruction. After incubation for 1–5 days at 37°C with 5% CO_2_, 10% CCK-8 solution was added to each well and incubated for 1 h. The absorbance value was measured at 450 nm using Multiscan GO (Thermo scientific).

### Wound Healing Assay

The wound healing assay was carried out to explore the role of miR-18a-5p in HRMECs function. The cells were seeded in 12-well plates (2.5 × 10^5^ cells per well) and grown to approximately 60% confluence. They were then transfected with agomir-18a-5p (50 nM) or NC and expanded until cells attained more than 90% confluence. A vertical scratch was created symmetrically across the confluent cell monolayer with a sterile 200-μL pipette tip. The floating cells and cellular debris were carefully removed by flushing them away with PBS and then replenishing each well with fresh culture medium. Closure of the denuded regions was monitored by capturing images of wound closure at 0 and 24 h observed with an inverted microscope (DMi8; Leica Microsystems Inc., Buffalo Grove, IL, United States). Cell migration areas were analyzed using ImageJ.

### Transwell Assay

Transwell migration assay was performed with 8-mm pore size culture inserts (Transwell; Corning, NY, United States) placing into the wells of 24-well culture plates. Four hundred microliters of ECM containing 10% NCS were added in the lower chamber of each well. 1.5 × 10^5^ cells (transfected with either miR-18a-5p or NC for 24 h) in 200 μl of DMEM were then added to the upper chamber. At 48 h after transfection, 1.5 × 10^5^ cells in 200 μl of NCS-free ECM were then added to the upper chamber. After 24 h of incubation, the cells that had migrated through the pores were fixed by 4% paraformaldehyde, stained with crystal violet (Beyotime Biotechnology, Shanghai, China) for 15 min, respectively. The images were taken immediately using a microscope, and the cells were counted by ImageJ.

### Tube Formation Assay

The HRMECs were plated in 6-well plates and transfected with 50 nM agomir-18a-5p or scrambled agomir (NC) for 48 h. The cells were harvested and cultured in a Matrigel Matrix (100 μl/well; Corning, NY) pre-coated 48-well plate (4 × 10^4^/well). After 6 h of incubation, the cells were photographed using an inverted microscope. The parameters of tube formation by HRMECs were measured by ImageJ with the Angiogenesis Analyzer plugin. The number of mesh and total tube length were quantified and showed in this study.

### Western Blot

Total proteins of HRMECs were extracted by RIPA Lysis Assay (Beyotime Biotechnology, Shanghai, China), and the concentration was measured by Pierce BCA Protein Assay Kit (Thermo Fisher Scientific, Rockford, IL, United States) according to the manufacturer’s instructions. Fifty micrograms of total proteins were separated in 15% sodium dodecyl sulfate (SDS) polyacrylamide gels and transferred onto polyvinylidene difluoride (PVDF) membrane. The membrane was blocked in 5% skimmed milk for 2 h and subsequently incubated overnight at 4°C with primary antibodies against FGF1 (1:200; Abcam, CA, United States), HIF1A (1:200; CST, Boston, MA, United States), or β-actin (1:2,000; Invitrogen, Rockford, IL, United States). Horseradish peroxidase (HRP)-linked anti-rabbit IgG (1:1,000; Cell Signaling Technology) was used as the secondary antibody. Specific protein bands were detected with Clarity^TM^ Western ECL Substrate Kit (Bio-Rad) and captured by FluorChem E system (Bio-Techne, Minneapolis, MN, United States). The gray intensity value of each protein band was analyzed by ImageJ.

### Luciferase Reporter Assays

The 3′ untranslated region (UTR) of human FGF1 was amplified and cloned into pmirGLO vector (Promega, Madison, WI, United States). The mutated seed region was generated by a site-directed mutagenesis method to remove complementarity to nucleotides of miR-18a-5p. HEK293 cells were cultured in 96-well plates and co-transfected with 100 ng/well of recombinant pmirGLO vector and 50 nM of miR-18a-5p or NC with Lipofectamine 2000 transfection reagent (Thermo Fisher Scientific). The luciferase activity was detected with Dual-Glo Luciferase Assay System (Promega) according to the manufacturer’s instructions.

### Statistical Analysis

All data were analyzed and plotted using GraphPad Prism software and presented as the mean ± standard error of the mean (SEM). Statistical differences were analyzed by Student’s *t*-test or One-way ANOVA for multiple comparisons of mean values. *p* < 0.05 was considered statistically significant.

## Results

### MiR-18a-5p Expression During Development and OIR Model

**FIGURE 1:**
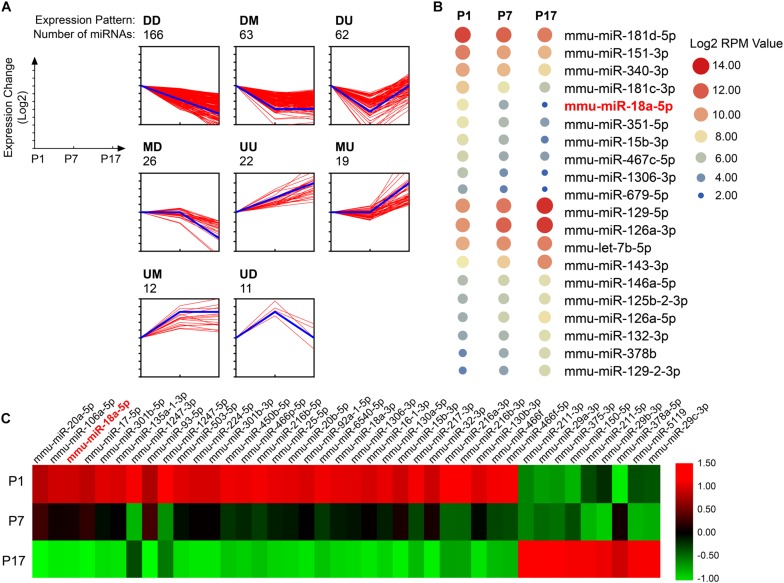
Dynamic expression profiles of miRNA during retinal development. **(A)** The expression patterns of miRNAs specifically regulated in developing retinas (*n* = 5 per group). The miRNAs were assigned to the model profile that more closely matched its time series according to the correlation coefficient. D, down-regulated; M, maintain; U, up-regulated. Thick blue line, model profile of log of expression change ratio over time. Thin red line, all individual gene expression profiles. **(B)** Twenty typical continuously down-regulated or up-regulated miRNAs. RPM, the number of reads per million (RPM) clean tags. **(C)** The 40 most differentially expressed miRNAs during retinal development from postnatal day 1 (P1) to P17.

**FIGURE 2:**
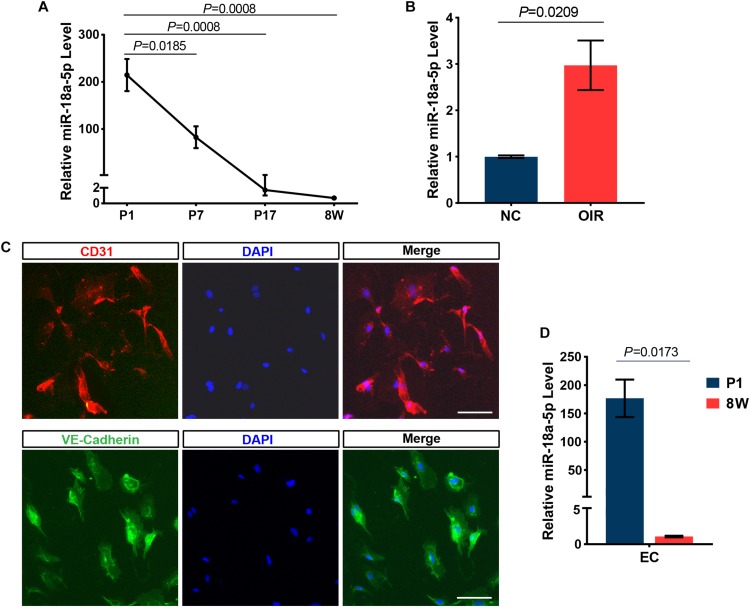
MiR-18a-5p expression during development and OIR model. **(A)** MiR-18a-5p was significantly down-regulated during developing retina (*n* = 5 per group; One-way ANOVA with Bonferroni correction). **(B)** The miR-18a-5p expression level was increased by approximately 3-fold in OIR mice (*n* = 6 per group; unpaired *t*-test). **(C)** The isolated RECs were identified by immunostaining using pan-endothelial markers anti-CD31 and anti-VE-Cadherin antibody. Scale bar, 50 μm. **(D)** MiR-18a-5p retinal endothelial cells (RECs) from 8-week-old (8W) mice was markedly down-regulated compared to newborn pups (*n* = 3 per group; unpaired *t*-test). OIR, oxygen-induced proliferative retinopathy; NC, normal control.

To identify miRNAs that are specifically regulated in developing retina, total RNAs isolated from pups retina at P1, P7, and P17, and performed high-throughput sequencing (GEO accession No. GSE142029). Based on the expression pattern analysis using STEM software, which implements a novel method for clustering short time series expression data that can differentiate between real and random patterns, miRNAs were mainly assigned to DD (down-regulated and down-regulated) profiles (Figure 1A). MiR-18a-5p was a typical miRNA that was consistently down-regulated during retinal development from P1 to P17 (Figure 1B). Moreover, miR-18a-5p was one of the most differentially expressed miRNAs (Figure 1C). Quantitative qPCR validated that miR-18a-5p was significantly down-regulated during retinal development (Figure 2A). Those the relative miR-18a-5p levels were decreased approximately 3-fold at P7, 125-fold at P17 and 300-fold at 8 weeks compared to P1 retina. On the other hand, the retinal miR-18a-5p levels were significantly up-regulated in OIR mice (Figure 2B). In order to verify that miR-18a-5p was expressed in the endothelium during retinal development, we have isolated RECs by MACS magnetic separation methods from retina and confirmed using pan-endothelial markers, anti-CD31 and anti-VE-Cadherin antibody (Figure 2C). In results, we showed that the miR-18a-5p expression levels in RECs of adult mice was significantly down-regulated compared to pups (Figure 2D), which is consistent with whole retinal expression pattern. Taken together, these results suggested that miR-18a-5p played a potential regulatory role in retinal physiologic and pathologic vascularization.

### MiR-18a-5p Suppressed Neovascularization in OIR Mice

**FIGURE 3:**
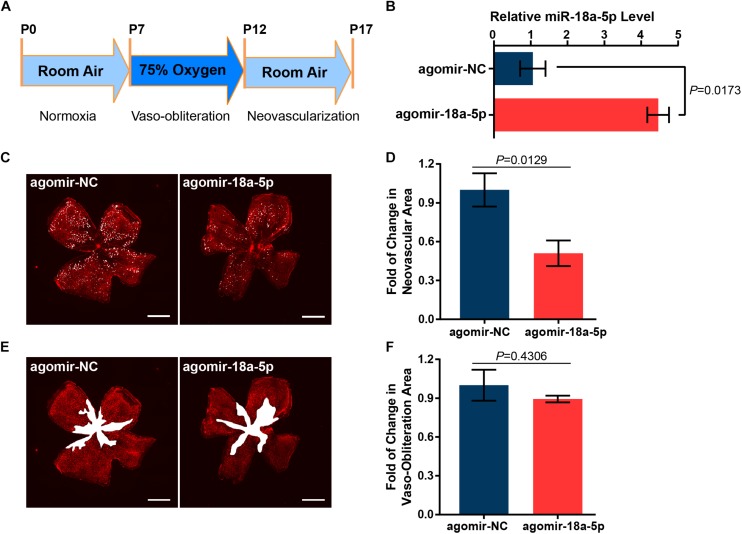
MiR-18a-5p suppressed pathologic neovascularization in OIR. **(A)** Schematic of establishment of OIR mice model. **(B)** The relative miR-18a-5p expression level in OIR mice with intravitreal injection of agomir-18a-5p or agomir-NC. **(C,E)** Representative images of P17 OIR retinas injected with agomir-18a-5p or agomir-NC. Pathologic neovascularization and vaso-obliteration were labeled as white spot and area, respectively. Scale bar, 500 μm. **(D,F)** Quantitative analysis of pathologic neovascularization and vaso-obliteration, respectively. The data are presented as mean ± SEM (*n* = 6 per group; unpaired *t*-test). OIR, oxygen-induced proliferative retinopathy.

To determine whether miR-18a-5p contributes to modulating retinal neovascularization, the OIR mice model was established (Figure 3A) and were treated with intravitreal injection of agomir-18a-5p (a type of chemically modified miR-18a-5p mimic) at P12. The expression level of miR-18a-5p at P17 was markedly increased in the agomir-18a-5p injected mice compared to scrambled agomir group (agomir-NC) (Figure 3B). At P17, agomir-18a-5p suppressed retinal neovascularization (∼50%; Figures 3C,D) but not vaso-obliteration (Figures 3E,F) compared with agomir-NC control. Thus, miR-18a-5p showed therapeutic effect in retinal neovascularization in OIR model.

### MiR-18a-5p Suppressed HRMECs Function

**FIGURE 4:**
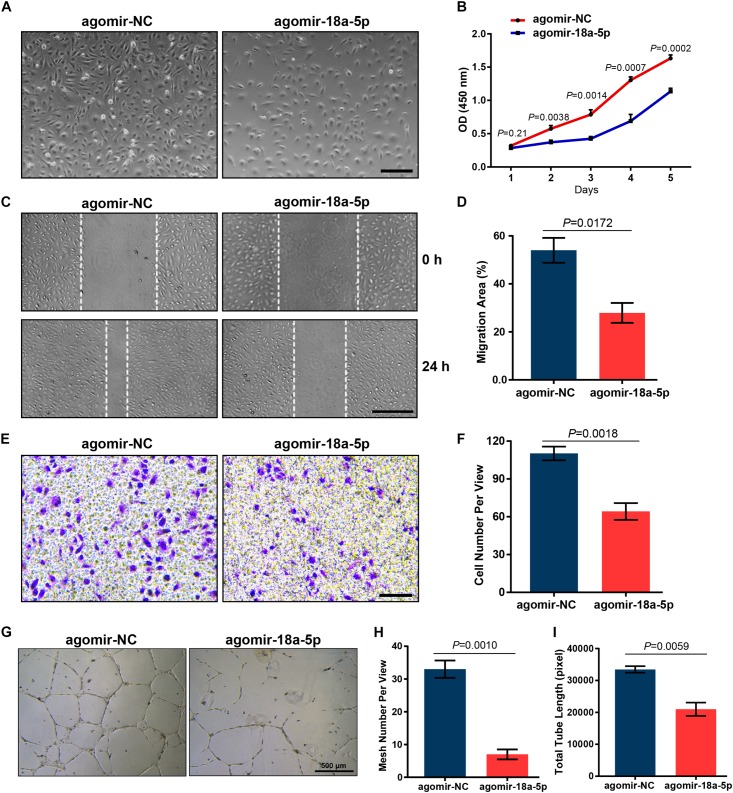
Effect of miR-18a-5p on HRMECs function. **(A)** Representative images of HRMECs transfected with agomir-18a-5p or agomir-NC. Scale bar, 200 μm. **(B)** HRMEC proliferation was suppressed by agomir-18a-5p treatment. The data are presented as mean ± SEM (*n* = 3 per group; unpaired *t*-test). **(C,E)** Agomir-18a-5p treatment significantly suppressed the migratory ability of HRMECs wound healing or transwell assay. Scale bars, 200 μm. **(D,F)** Quantitative analysis for HRMECs wound healing and transwell assay. The values are presented as mean ± SEM (*n* = 3 per group; unpaired *t*-test). **(G)** Representative images of tube formation assay in HRMECs with agomir-18a-5p or agomir-NC transfection. Scale bar, 500 μm. **(H,I)** Quantitative analysis of HRMECs in mesh numbers and total tubule length. The data are presented as mean ± SEM (*n* = 3 per group; unpaired *t*-test). HRMECs, human retinal microvascular endothelial cells; CCK-8, Cell Counting Kit-8.

To further explore the inhibitory effect of miR-18a-5p on endothelial function, agomir-18a-5p was transfected into HRMECs and then evaluated its effect on cell proliferation and migration. The CCK-8 proliferation assay revealed that the cells treated with agomir-18a-5p showed significant reduction in absorbance compared with agomir-NC (Figure 4A). There was a significant decrease in cell number from day 2 to day 5 (Figure 4B), which indicated that agomiR-18a-5p inhibited the viability of HRMECs. We then conducted wound healing and transwell assay to detect the migratory capacity of HRMECs with agomir-18a-5p transfection. The agomir-18a-5p transfected cells showed 48% reduction in wound healing (Figures 4C,D) and 42% in transwell assay (Figures 4E,F) compared with agomir-NC. These results suggest that ectopic expression of miR-18a-5p restrains HRMECs migratory activity. To further evaluate the angiogenic effect of miR-18a-5p on HRMECs, we conducted the tube formation assay. As shown in Figure 4G, agomir-18a-5p substantially suppressed the tube formation of HRMECs, resulting in reductions of 73% in mesh numbers (Figure 4H) and 37% in total tubule length (Figure 4I). Taken together, miR-18a-5p up-regulation restrains the normal function of HRMECs including proliferation, migration, and the tube formation ability.

### MiR-18a-5p Targeted Angiogenic Genes FGF1 and HIF1A

**FIGURE 5:**
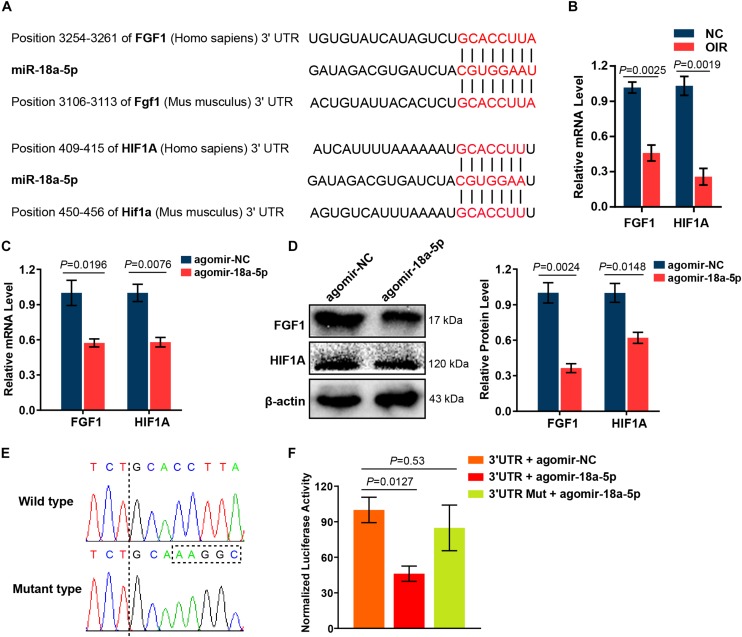
MiR-18a-5p targeted angiogenic genes FGF1 and HIF1A. **(A)** Alignment between the binding sites of miR-18a-5p and its targets. **(B)** FGF1 and HIF1A mRNA levels in OIR retinas at P17 (*n* = 5-8 per group; unpaired *t*-test). **(C)** FGF1 and HIF1A mRNA levels in HRMECs with agomir-18a-5p or agomir-NC transfection (*n* = 3 per group; unpaired *t*-test). **(D)** The protein levels of FGF1 and HIF1A in HRMECs with agomir-18a-5p or agomir-NC transfection (*n* = 3 per group; unpaired *t*-test). **(E)** Sequencing results of pmirGLO-FGF1 3′ UTR and pmirGLO-FGF1 3′ UTR-Mut. **(F)** Luciferase activity of HEK293 cells co-transfected with pmirGLO-FGF1 3′UTR or pmirGLO-FGF1 3′ UTR-Mut, and agomir-18a-5p or agomir-NC (*n* = 3 per group; unpaired *t*-test). FGF1, fibroblast growth factor 1; HIF1A, hypoxia-inducible factor 1-alpha; HRMECs, human retinal microvascular endothelial cells; HEK293, human embryonic kidney 293; NC, normal control; 3′ UTR, 3′ untranslated region; Mut, mutant.

It has been reported that the effect of miRNAs on the endothelial cell function and vessel growth can be elucidated by regulating their target genes. To identify potential target genes of miR-18a-5p, we analyzed the seed sequence of miR-18a-5p (CGUGGAA), conserved in both human and murine subjects. These bases were complementarily paired with the 3′ untranslated region (UTR) of FGF1 and HIF1A (Figure 5A). The mRNA levels of FGF1 and HIF1A in OIR retina were markedly decreased at P17 compared with normal control (NC) group (Figure 5B). Moreover, overexpression of miR-18a-5p in HRMECs significantly reduced the FGF1 and HIF1A expression both in mRNA and protein levels (Figures 5C,D). HIF1A has been identified as a target of miR-18a-5p (Krutilina et al., 2014). Consequently, luciferase reporter assay was carried out to determine the FGF1 is a novel target gene of miR-18a-5p. The wild-type or mutant 3′ UTRs of FGF1 was successfully cloned into the luciferase reporter vector pmirGLO (Figure 5E). The result of the luciferase reporter assay revealed that agomir-18a-5p transfection significantly reduced the luciferase activity compared to the agomir-NC group (Figure 5F). Whereas, that the mutant reporter vector abolished the interactions between agomir-18a-5p and FGF1 3′ UTRs. Our finding suggests those roles of miR-18a-5p in EC function and retinal neovascularization are through inhibiting the direct target genes FGF1 and HIF1A.

## Discussion

VEGF is a potent proangiogenic factor. Anti-VEGF drugs have been used to treat ocular neovascularization with certain efficacy. However, anti-VEGF therapy does have some limitations and may cause undesirable side effects. Several articles have reported that VEGF is necessary for vascular homeostasis (Carmeliet et al., 1996; Lee et al., 2007). Long-term clinical application of anti-VEGF drug may result in adverse ocular responses (Falavarjani and Nguyen, 2013). Due to continuous development of novel drugs, it will become more difficult to prove superiority of one drug versus another (Grossniklaus et al., 2010). However, it is still necessary to find new drugs to treat neovascular eye diseases as supplements to or even possible replacements for anti-VEGF drugs.

Non-coding RNAs (ncRNAs), including miRNA, long non-coding RNA (lncRNA) and circular RNA (circRNA), are related not only to tumorigenesis, but also to neurological, cardiovascular, developmental, and other diseases (Esteller, 2011). Dozens of miRNAs have been used as diagnostic and prognostic biomarkers, and some miRNAs intended to treat diseases as varied as cancer, hepatitis, and scleroderma have reached clinical trials (Maurer et al., 2010; Bader, 2012; Janssen et al., 2013). It is easier to design and synthesize oligonucleotide-based drugs than to identify small molecules, and it makes the process of identifying active ncRNA drugs much faster than that of small-molecule drugs (Matsui and Corey, 2017; Nakamori et al., 2019). To utilize the mammalian RNAi pathway for potent and specific inhibition of putative therapeutic targets, RNAi drugs have the same advantages (Setten et al., 2019). To date, few RNAi-based drugs have received approval. Examples include Vitravene being applied to treat ocular/CMV retinitis (Roehr, 1998), and Kynamro being developed to treat systemic/familial hypercholesterolemia (Geary et al., 2015). Despite considerable progress, there are still some critical challenges in the development of oligonucleotide-based therapeutics, including identification of the best oligonucleotide drugs and delivery methods for each disease type, avoidance of non-specific toxicity caused by immunogenic reactions, off-target effects, and renal accumulation (Rupaimoole and Slack, 2017; Levin, 2019; Setten et al., 2019).

Recent studies have shown that multiple miRNAs were aberrantly expressed in pathological vessel growth and played vital roles in regulation of the neovascular eye diseases. Several specific miRNAs were highly expressed in ECs that curbed angiogenesis. These anti-angiogenic miRNAs include miR-126, miR-150, miR-184, and miR-342, whereas miR-132, miR-27, miR-155, and members of the let-7 family promoted angiogenesis (Zhang et al., 2017). The miR-17-92 cluster is one of the most extensively studied miRNAs, especially in tumor angiogenesis. Overexpression of the miR-17-92 cluster was shown to promote tumor angiogenesis (Dews et al., 2006). Transfection of ECs simultaneously with miR-18a, miR-17-5p, and miR-20a also rescued the defect in endothelial cell proliferation and morphogenesis initiated by the loss of Dicer (Suarez et al., 2008). On the contrary, Doebele et al. found that members of the microRNA-17-92 cluster exhibited a cell-intrinsic antiangiogenic function in ECs, and antagomir-17/20 selectively enhanced neovascularization of Matrigel plugs but did not affect tumor angiogenesis (Doebele et al., 2010). Based on the above information, the regulation of angiogenesis by miR-17-92 cluster is complex, and specific members of this cluster may mediate differential and context-dependent effects on neovascularization. It seems more reasonable to target individual members of the miR-17-92 cluster to gain an accurate and deep understanding of their role in angiogenesis modulation.

In this study, miR-18a-5p was shown to be highly expressed in embryonic retinas and significantly down-regulated during the course of development. On the other hand, the expression level of miR-18a-5p in OIR retinas was higher than that of normal retinas. It can be speculated that miR-18a-5p may be involved in the initiation and progression of angiogenesis. Therefore, we evaluated the potential role and anti-angiogenic efficacy of miR-18a-5p in treating retinal neovascularization. Our study showed that the upregulation of miR-18a-5p was capable of inhibiting pathological angiogenesis in an OIR model as well as HRMEC functions. Accumulating evidence shows that FGF1 and HIF1A are closely associated with angiogenesis, which are target genes of miR-18a-5p.

FGF1 belongs to the FGF family and exerted a pro-angiogenic effect on ECs (Ding et al., 2003; Mori et al., 2013). FGF1 and VEGF have been demonstrated to be synergistic in the induction of angiogenesis as exposure of ECs to both FGF1 and VEGF in combination stimulated a greater angiogenic response than the response to either growth factor alone (Xue and Greisler, 2002). FGF1 also contributed to the increased number of blood vessels in the middle ear mucosa during otitis media (Husseman et al., 2012). In addition, controlled delivery of FGF1 and neuregulin-1 (NRG1) from biodegradable microparticles could promote cardiac repair in a rat myocardial infarction model through induction of tissue revascularization and activation of endogenous regeneration (Formiga et al., 2014).

HIF1A is an important transcription factor that tends to be activated in the hypoxia condition, in which it is known to increase the expression of VEGF and promote angiogenesis (Shweiki et al., 1992; Jones et al., 2002; Ahluwalia and Tarnawski, 2012). HIF1A was also correlated to other genes in the angiogenesis pathway such as FGF2, and platelet-derived growth factor alpha (PDGFA) (Hoffmann et al., 2008). Non-steroidal anti-inflammatory drugs could inhibit hypoxia-induced *in vitro* angiogenesis in gastric microvascular ECs via reducing accumulation of HIF1A (Jones et al., 2002). Previous studies have revealed that HIF1A was overexpressed in multiple types of human cancer. Overexpression of HIF1A and HIF2A in squamous cell head-and-neck cancer (SCHNC) was related to locally aggressive behavior, intensification of angiogenesis, and resistance to carboplatin chemoradiotherapy (Koukourakis et al., 2002). There was a significant correlation between microvessel density and HIF1A expression in non-myoinvasive endometrioid carcinomas as well (Espinosa et al., 2010).

In the present study, overexpression of miR-18a-5p in HRMECs could significantly reduce the mRNA and protein levels of FGF1 and HIF1A. Studies also confirmed that miR-18a-5p is a direct regulator of these genes in ECs through targeting their 3′ UTR seed sequences. Therefore, miR-18a-5p might function as a suppressor of retinal neovascularization by targeting these two genes. Our study provides evidence of the association between miR-18a-5p and pathologic retinal angiogenesis. MiR-18a-5p is a potential therapeutic target for treatment neovascular ocular diseases.

## Data Availability Statement

The datasets generated for this study can be found in NCBI GEO accession GSE142029.

## Ethics Statement

All animal experiments were performed strictly according to the ARVO Statement for the Use of Animals in Ophthalmic and Vision Research and approved by the Animal Care and Use Committee of the Wenzhou Medical University.

## Author Contributions

Z-LC, RD’A, JQ, and FL conceptualized and designed the study. J-TG, X-XL, D-WP, and W-MZ acquisition of the data. J-TG, X-XL, D-WP, and Z-LC analyzed and interpreted the data. J-TG, D-WP, and Z-LC wrote, reviewed, and/or revised the manuscript. All authors have read and approved the final version of the manuscript.

## Conflict of Interest

The authors declare that the research was conducted in the absence of any commercial or financial relationships that could be construed as a potential conflict of interest.
